# Plant *in situ* tissue regeneration: dynamics, mechanisms and implications for forestry research

**DOI:** 10.48130/FR-2023-0008

**Published:** 2023-03-30

**Authors:** Yufei Zhang, Xiaoyu Wang, Jing Zhang, Xin-Qiang He

**Affiliations:** 1 State Key Laboratory of Protein and Plant Gene Research, School of Life Sciences, Peking University, Beijing 100871, China; 2 Department of Plant Biology and Ecology, College of Life Sciences, Nankai University, Tianjin 300071, China

**Keywords:** Plant *in situ* regeneration, Secondary vascular tissue regeneration, Plant apex regeneration, Tissue reunion, Woody plant development

## Abstract

Plants possess remarkable developmental plasticity and regenerative ability to reshape themselves in response to external stimulations. After localised injuries, they can initiate cellular reprogramming at the wound sites to repair or regrow structures that could substitute the functionality of the damaged or lost parts. This way of regeneration in plants is called plant *in situ* tissue regeneration. Upon wounding like excision, incision or girdling, the original tissue patterns are completely or partially destroyed, the remanent tissues could perceive the wounding signals and thereby initiate cell de-differentiation, trans-differentiation or re-differentiation to reconstruct the lost or damaged tissues. In this review, we summarize the regenerative dynamics and regulatory mechanisms during the major *in situ* tissue regeneration processes in plants, including secondary vascular tissue (SVT) regeneration after girdling, apex regeneration after excision and tissue reunion after incision. In addition, we compare the features of SVT regeneration, the most relevant system for forestry, with other plant *in situ* tissue regeneration systems. We further discuss the unsolved issues and the potential applications of plant *in situ* regeneration for forestry research, aiming to provide new insights for the study of woody plant development.

## Introduction

Regeneration refers to the process by which the tissues or organs of an organism repair or replace themselves after being damaged. In animals, some species, such as *Hydra vulgaris* and* Schmidtea mediterranea*, can regenerate at the wound sites after cutting part of their body^[1]^. Plant cells are totipotent and have a more powerful regenerative capability than animal cells. Therefore, plant regeneration has been widely used in forestry and horticulture production and research. For example, propagation by stem cutting is a rapid way to propagate many trees^[2]^ and grafting can help plants to gain new advantageous traits^[3−5]^. Roots and shoots can regenerate *de novo* from a cut piece of tissue in the medium, which is the basis of plant tissue culture^[6−8]^. Plant regeneration can be generally divided into three types^[1]^. The first type is tissue repair or *in situ* regeneration, which means that plants restore damaged or lost organs or tissues after local injury. For example, root or shoot can repair or regrow structures capable of restoring the original tissues’ functions after excision^[9,10]^. The second type is *de novo* organogenesis, which means that plant explants regenerate new tissues or organs that did not exist previously. For example, root explants can be induced to form new shoots under certain conditions^[8]^. The third type is somatic embryogenesis, which means that an individual somatic cell obtains totipotency and regrows into an entire plant^[11,12]^.

**Figure 1:**
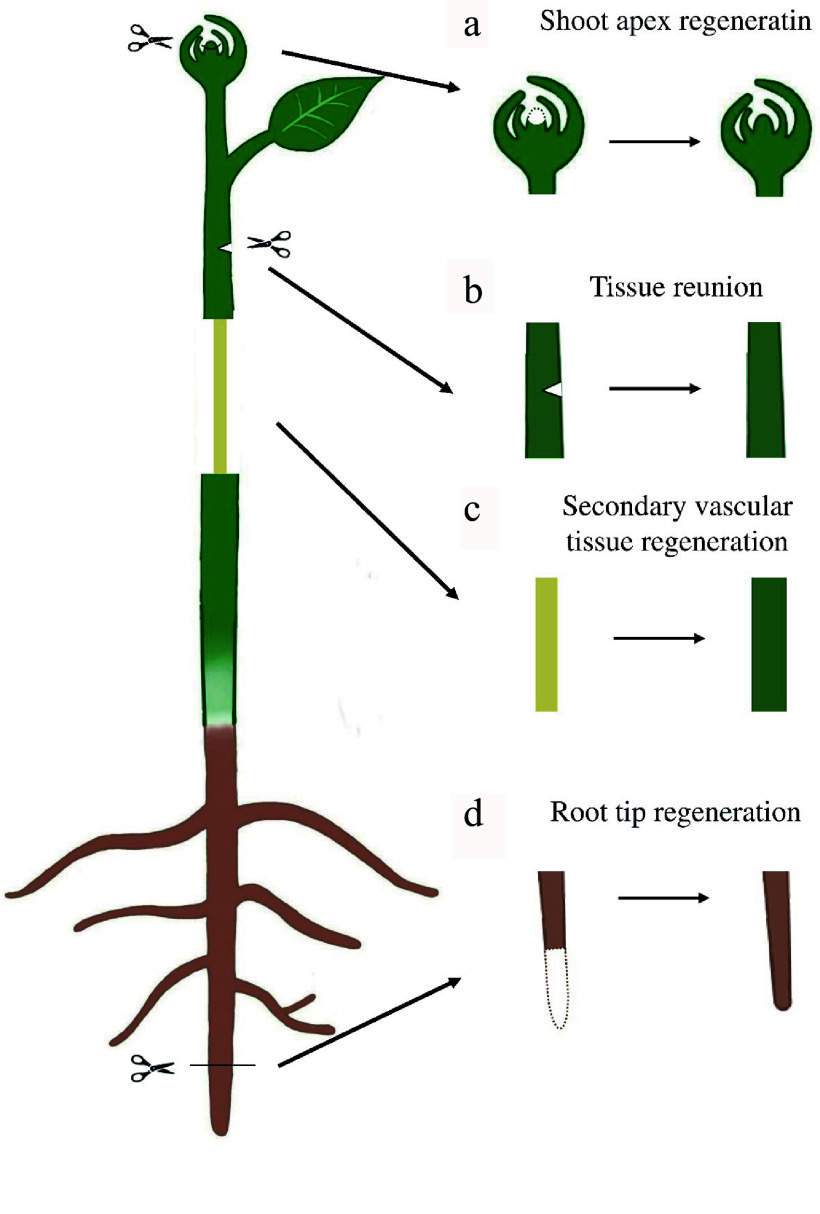
Diverse types of plant *in situ* tissue regeneration systems. (a) Shoot apex regeneration after excision. (b) Tissue reunion after incision. (c) Secondary vascular tissue (SVT) regeneration after bark girdling. (d) Root tip regeneration after excision.

There are various *in situ* tissue regeneration systems in plants (Fig. 1) and the most studied ones include apex repair after excision^[13]^, tissue reunion after incision^[14]^ and secondary vascular tissue (SVT) regeneration after girdling^[15]^. The apex regeneration is the most understood plant *in situ* tissue regeneration system. When part or the whole apex in a plant is excised, the remained surrounding cells are reprogrammed to reconstruct a new shoot apex (Fig. 1a) or root tip (Fig. 1d) containing shoot or root apical meristem^[16−18]^. Tissue reunion after incision (Fig. 1b) usually involves vascular and ground tissue re-connection at the injury site to restore the capacity for mechanical support and continuity of vasculature for transport in plant hypocotyl or inflorescence stem^[14,19,20]^. Among these systems, SVT regeneration (Fig. 1c) is of the greatest interest for forestry research due to the relevance to wood production and forest conservation. SVT system consists of secondary xylem or wood, secondary phloem and the meristem that gives rise to them, vascular cambium^[21]^. After girdling, tissues on bark side including periderm, secondary phloem or vascular cambium are removed, remarkably, the remained differentiating xylem cells can regenerate a new bark including phloem and functional vascular cambium^[15]^.

Earlier and recent studies have described the morphological and physiological changes in all the regeneration systems and with biochemical, genetical and molecular approaches, regulatory mechanisms such as transcriptional regulation and hormonal regulation are uncovered^[14,16,17,22]^. Thanks to the development of plant genomic and gene editing technologies as well as single-cell RNA sequencing (scRNA-seq) in recent years, we have a deeper understanding of the molecular mechanisms in plant regeneration^[23−25]^. In this review, we summarize the current findings discovered in the major plant *in situ* tissue regeneration systems and compare the SVT regeneration system, which is the most relevant to forestry research, with other plant *in situ* tissue regeneration systems. We also discuss the problems that need to be resolved regarding plant *in situ* regeneration and the implications for forestry research, aiming to provide new ideas for the study of woody plant development.

## Regenerative dynamics in plant *in situ* tissue regeneration

Using *Arabidopsis thaliana*, *Populus* spp. (poplar) and other plant species as models, researchers have demonstrated the regenerative processes on the tissue or cellular level in different plant *in situ* regeneration systems. Herein we mainly describe the regenerative responses in the three major regeneration systems, highlighting the SVT regeneration, the most relevant system for forestry studies.

### SVT regeneration after girdling

Bark removal is a tool used in practical and basic research^[26]^. In horticulture, bark removal in a small area has been applied to promote early flowering and fruit production^[27]^. Girdling of sick bark helps to eliminate pests and diseases^[28]^. Large scale girdling in *Eucommia ulmoides* (*E. ulmoides*) can provide continuous production of the bark for medical use^[29]^, and this system has been also explored in other tree species, including *Broussonetia papyrifera*, *Betula pubescens*, *Malus pumila, Ginkgo biloba* (*G. biloba*) and poplar. However, the regenerative patterns and efficiency are dependant on the tree species^[30−32]^. For example, in *G. biloba*, vascular cambium is reconstructed from callus rather than the differentiating xylem and this process takes up to one month compared to 5−7 d in *E. ulmoides*^[30,32]^. In addition, SVT regeneration can also occur in herbaceous plants with vigorous xylem such as *Solanum melongena* and *Helianthus tuberosus *(*H. tuberosus*), however, the regenerative speed and process are not exactly the same between woody plants and herbaceous plants. Generally, the vascular tissues of herbaceous plants regenerate faster than woody plants and the morphology of regenerated tissues is different^[33,34]^. For example, the activity of regenerated cambium in *H. tuberosus* is weak, resulting in regeneration of irregularly arranged vascular bindles and uneven bark in the later stage, unlike in trees as *E. ulmoides*, the surface of the regenerated bark is as smooth as the original bark^[34]^.

**Figure 2:**
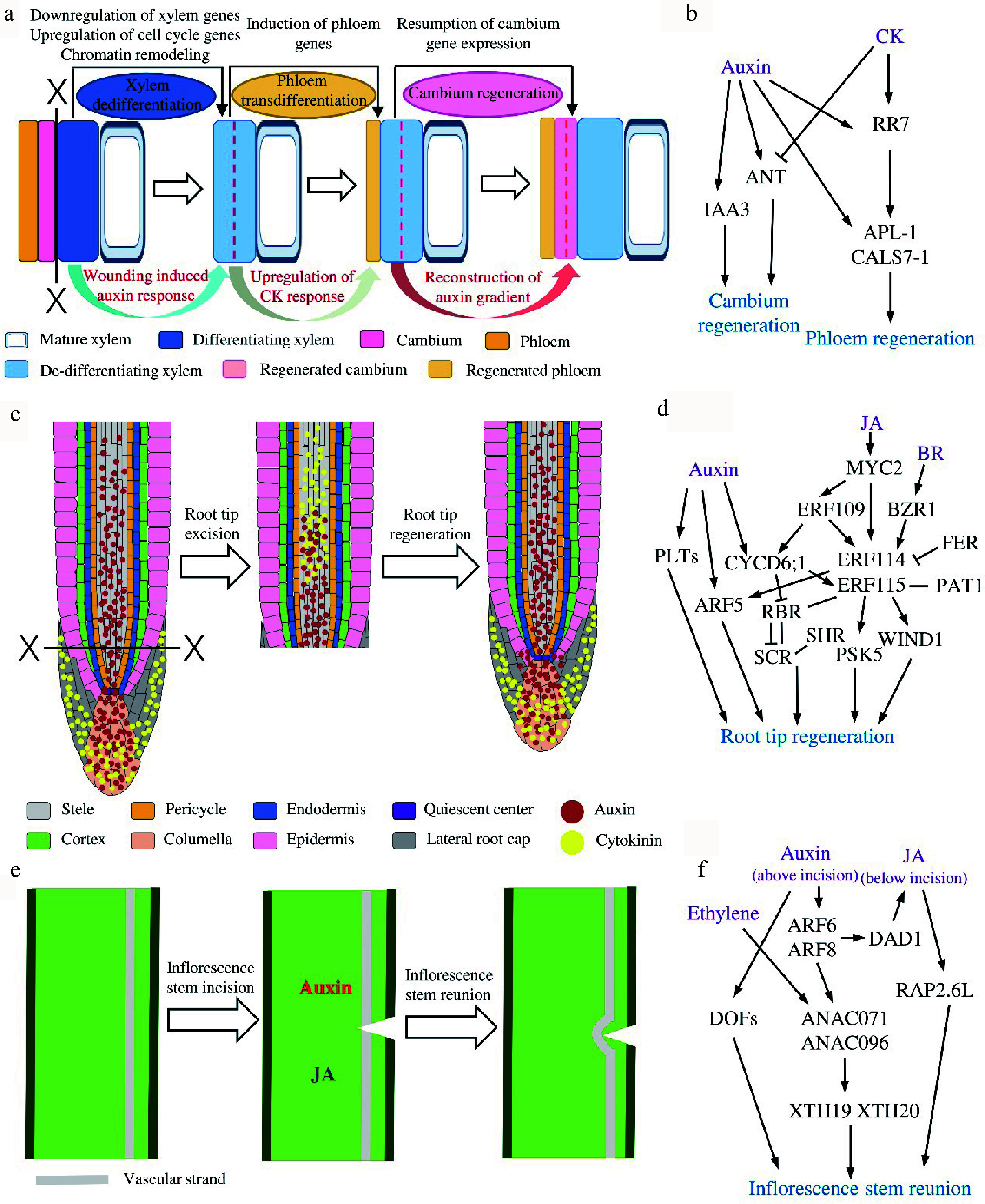
The regenerative dynamics and regulatory models of three plants *in situ* tissue regeneration systems. (a) Secondary vascular tissue (SVT) regeneration after bark girdling and (b) the proposed regulatory model in this system. Dotted lines indicate cell division. (c) Root tip regeneration after excision and (d) the regulatory model in this system. (e) Inflorescence stem reunion after incision and (f) the regulatory model in this system.

SVT regeneration after girdling in poplar and *E. ulmoides* can be generally divided into three stages^[15,22,26,35]^ (Fig. 2a). Upon bark girdling, tissues including periderm, secondary phloem and vascular cambium are usually removed, leaving the differentiating xylem and mature xylem on the trunk. At the first stage, the differentiating xylem cells regain the ability to divide under appropriate conditions. The differentiating tracheary elements or fibers undergo periclinal and transverse divisions to de-differentiate, while ray cells expand and divide rapidly to form callus, covering the surface of the trunk at 2−6 d after girdling (DAG)^[22,35]^. At the second stage, take poplar as an example, the newly formed sieve elements (SEs) could be detected with aniline blue, a callose specific fluorescent dye^[36]^, at 6−9 DAG, near the de-differentiating xylem cells but not in callus. Autoradiography with [^14^C]-sucrose revealed that the regenerated SEs could regain the transport ability which was blocked by girdling. The fact that regenerated SEs with typical morphology and function of phloem sieve tube members occur prior to the resumption of functional cambium suggests that the regenerated phloem cells trans-differentiate from xylem cells^[22,35]^. At the third stage, a continuous cambium can be found at around 12 DAG in poplar and 21 DAG in *E. ulmoides*. In poplar, discontinuous flat cambium cells could be seen on the inside of SEs at 9 DAG and a continuous cambium with ray initial and fusiform initial cells is basically established around 12 DAG. Short tracheary elements could be observed at the same stage. In addition, the number of SEs is increased and stone cells with thickened cell wall are found in phloem. This reveals that the regenerated cambium becomes functional to differentiate into new secondary phloem outward and secondary xylem inward^[22,26]^. At the same time, phellogen-like cells are also found in out layers of callus that could differentiate into cork cells and reconstitute a functional periderm^[15]^.

### Plant apex regeneration after excision

Plant apices contain the meristems including shoot apical meristem (SAM) and root apical meristem (RAM) that are responsible for the primary growth of plants. Therefore, the repair of SAM or RAM after wounding is crucial for plant survival and the related research has always been a hot topic in the field of plant science. As early as the 1950s, researchers found that the shoot apex could regenerate after the excision operations in *Solanum tuberosum* (potato) and *Lupinus albu* (white lupine)^[37,38]^. In white lupine, cutting the middle area of the shoot apex could induce the initiation of new apices on both sides of the wounded site and the newly formed apices would gradually replace the original damaged apex to produce new shoots^[37]^. It is known that SAM is the source of organs above the ground in plants and it consists of L_1_, L_2_ and L_3_ layers. The central zone (CZ) and peripheral zone (PZ) are arranged transversely on the L_1_ to L_3_ layers. The CZ contains undifferentiated stem cells that can divide continuously, allowing the surrounding cells to enter the PZ, which is responsible for lateral organ formation^[8,39]^. With the tissue-specific ablation, we now have a better understanding on the regeneration of SAM and the roles of each layer within SAM. After laser ablation of the CZ in the SAM of *Solanum lycopersicum* (tomato)^[18]^, the wound is closed and displaced gradually with a new CZ forming in the meristem. The removal of CZ does not affect the formation of new organs, indicating that CZ has no direct effect on organogenesis. The removal of PZ affects the positioning of new leaves, but does not affect the function of CZ. The ablation of L_1_ in the CZ region changes the pattern of cell division, leading to cell stacks at the cut surfaces of SAM. Intriguingly, removing the entire L_1_ causes the failure of regeneration and organ formation, suggesting that L_1_ controls cell division direction and organ formation^[18]^.

Root tip regeneration is the most extensively studied and well understood plant* in situ* regeneration system. Studies in *Zea mays* (maize)^[40]^, *Pisum sativum *(pea)^[41]^, Arabidopsis^[17,42]^ and *Oryza sativa* (rice)^[43]^ all revealed a rapid regeneration of root tip after excision at appropriate positions. Commonly, root tip excision results in removing of quiescent center (QC), stem cells with their recent progenies in RAM and root cap. It is commonly accepted that multiple cell types are responsible for root tip regeneration, however, their contributions are different as demonstrated by lineage tracing experiments in Arabidopsis^[17,43]^. For instance, cells in outer layer doesn’t contribute to root tip restoration; endodermal cells mainly contribute to the reformation of epidermis and lateral root cap (LRC); xylem pole pericycle and protoxylem cells mostly produce new ground tissues including cortex and endodermal cells; cells in stele could change to all cells in root tip except epidermal and LRC cells. Importantly, almost all remnant cells around the wound are able to switch their identities to new stem cells by 24 h post-cut (HPC) and undergo stem-cell like divisions. Notably, functional recovery of columella is also displayed from 24 HPC, preceding the resumption of the QC, indicating that root tip regeneration does not require a functional stem cell niche^[42]^. Restoration of distal tissues such as endodermis, epidermis and stele occur within 48 HPC. Full re-establishment of the whole root tip could be completed in 3−4 d.

When root cap including columella is excised alone in rice or maize, it can regenerate within 72 h and starch granule accumulation could be detected in new columella as early as 24 h after excision^[43,44]^, on the same time scale as seen in Arabidopsis root tip^[42]^. In this process, the existence of QC and supply of auxin is required for successful root cap reformation^[43,44]^. Using laser-assisted cell ablation technique, researchers are able to eliminate certain types of cells precisely in the root tip. Targeted ablation of QC in roots leads to the re-specification of QC and root cap from the distal vascular cells^[45]^. On the other hand, when columella stem cells are abolished, QC cells start to divide to reconstruct a new stem cell domain^[45]^. Ablation of meristematic cells in RAM induces the periclinal cell divisions in the inner adjacent cells to regain the new stem cell identities and final restoration of the lost cells^[46]^. For example, after the cortex cell is ablated, the endodermis cells on the inside divide to form new cortex cells to fill the damaged position^[47]^.

### Tissue reunion after incision

The aboveground organs in plants are prone to mechanical damage in nature and injury like incision usually interrupts the tissue connection, particularly vascular connection between upper and lower parts in hypocotyls or stems. A similar scenario is also found in grafting that is widely used in horticulture and basic research. In general, during tissue reunion after partial incision, vascular and other cells are re-specified to connect physiologically to the existing tissues. The morphological dynamics of tissue reconstruction after local incision has been characterized in multiple plant systems. One day after the hypocotyl of 7-d-old *Cucumis sativus* (cucumber) or tomato is transversely incised to half the diameter, a cell wall-like structure appears at the wounded surface. At 3−5 d after incision (DAI), the cortex cells around the wound divide and elongate in an intrusive manner to fill the wounded region. A layer of cell wall forms at the surface where cortex cells attach the above and below region at the wound. No more cell division and elongation are observed after 7 DAI, indicating the full reunion of hypocotyl^[14,19]^.

Like the hypocotyl, the stem can also reconnect after incision under certain conditions (Fig. 1b). Deep incision in the stem disrupts vascular strand connection, thus prevents the transport of water and nutrients in the stems. The incision also triggers cell proliferation in various types of cells around the wound and successive stem reunion. After partial incision is performed in the Arabidopsis inflorescence stem, mainly pith and cortical cells neighboring the wound resume proliferation and intrusive elongation toward the cut surface from 3 DAI. However, it seems that cell divisions mostly occur above the wound and mainly pith cell divisions contribute to the vertical tissue connections^[14,20]^. A recent study using 3D image re-construction in the upper incision region further reveals that in addition to pith cells, parenchyma cells of protoxylem near the incision also divide asymmetrically thus contributing to tissue reunion^[48]^. Interestingly, vascular cambial activity is induced in parenchyma cells undergoing anticlinal division around the wound as well as in periclinal dividing cells within the upper swelling region at 3−7 DAI, leading to the re-differentiation of secondary xylem around and above the incision^[48]^. Such cambium-like cell divisions could provide cells in wound tissues for stem restoration at 7−10 DAI^[14,48]^. Notably, there are different regenerative paths for vascular reconnection after stem incision^[49]^. In plants with only primary tissue structures, xylem vessels and SEs are regenerated from de-differentiated parenchyma cells and they are arranged either around the wound or form the 'bypass' strands through the wound or bridges between the vascular bundles to connect the damaged body parts^[48−53]^. When cambial activity and secondary growth is induced prior to incision in Arabidopsis stem, complete vascular reconstruction occurs mainly above or around a wound (Fig. 2e) from 6 DAI as a consequence of cambial restoration and occasionally through bypass vessel strands differentiated from callus inside the wound between 10 to 13 DAI^[52]^. In woody plants with pronounced secondary growth and cambial activity, tissue reunion is accompanied by anticlinal divisions of cambial cells and their intrusive growth, which leads to the regeneration of vasculature in the incised regions^[49]^.

## Regulatory mechanisms in plant *in situ* tissue regulation systems

Earlier studies in varied plant systems including woody plants have uncovered the morphological changes of tissue repairs during these *in situ* regeneration processes. However, due to the limitations of histological analyses, only the final effects of regeneration could be observed and the dynamics on the cellular and molecular levels are lacking. With the help of cell biological, molecular, genetic and computational approaches particularly with the employment of model plants such as Arabidopsis, our understanding of the mechanisms underlying the *in situ* regeneration has increased rapidly. Cellular responses during the *in situ* regeneration commonly include cell proliferation, cell fate re-specification and cell re-differentiation. Evidence from a range of research has demonstrated that phytohormones and transcriptional regulators play central roles in each step. We summarise here the molecular regulators in the three major regeneration systems (Table 1).

**Table 1:** Roles of molecular regulators in plant* in situ* regeneration systems.

Regeneration systems	Regulators	Description	Roles in plant *in situ* regeneration	References
**SVT regeneration after girdling**	Auxin	Phytohormone	Inducing secondary vascular tissue (SVT) regeneration and required for cambium re-establishment	[22,26,31,35]
	Cytokinin (CK)	Phytohormone	Promoting phloem regeneration, inhibiting vascular cambium restoration in *in vitro* system by blocking auxin maximum	[22, 26]
	PtIAA3	AUX/IAA	Auxin responsive and induced during SVT regeneration	[26]
	PtRR7	Type-A response regulator	CK responsive and induced during SVT regeneration	[26]
	PtANT	AP2/ERF	Induced during cambium regeneration	[22, 26]
	PtAPL	G2-like	Induced during phloem regeneration	[22, 26]
	PtCALS7	Callose synthase	Induced during phloem regeneration	[22, 26]
**Root** **tip regeneration**	Auxin	Phytohormone	Accumulating at the wounding regions first and then moving distally, and required for the reconstruction of root apical meristem (RAM)	[17]
	CK	Phytohormone	Overlapping with auxin first and then moving proximally, and required for the reconstruction of RAM	[17]
	Jasmonic acid (JA)	Phytohormone	Stimulating stem cell niche regeneration in RAM	[16]
	PLTs	AP2/ERF	Promoting root quiescent center (QC) re-establishment and root tip regeneration, and determining regenerative potential	[71, 76]
	YUCs	Auxin synthesis	Required for root tip regeneration	[72]
	ARF5	ARF	Activated by auxin to promote the reconstruction of root tip	[10, 42]
	ERF115	AP2/ERF	Activated by JA, auxin and brassinosteroid (BR) to promote root regeneration after excision or cell ablation	[16, 73−75]
	ERF114	AP2/ERF	Acting redundantly with ERF115 to promote root tip regeneration	[75]
	ERF109	AP2/ERF	Activated by JA to promote the reconstruction of root tip	[16]
	PAT1	GRAS	Interacting with ERF115 to promote root tip regeneration	[73]
	WIND1	AP2/ERF	Regulated by ERF115 and required for root tip regeneration	[73]
	PSK5	Phytosulfokine	Regulated by ERF115 and required for root tip regeneration	[74, 75]
	SCR	GRAS	Promoting QC and cortex cell re-establishment after cell ablation	[47, 76]
	SHR	GRAS	Promoting QC and cortex cell re-establishment after cell ablation	[47, 76]
	FEZ	NAC	Promoting lateral root cap (LRC) cell re-establishment after cell ablation	[47]
	SMB	NAC	Promoting LRC cell re-establishment after cell ablation	[47]
**Tissue reunion after incision**	Auxin	Phytohormone	Accumulated above the incision and enhancing JA production to promote inflorescence stem reunion but not cucumber or tomato hypocotyl reunion	[20, 64]
	JA	Phytohormone	Produced below the incision to promote inflorescence stem reunion	[20, 64]
	Ethylene	Phytohormone	Interacting with auxin and promoting inflorescence stem reunion	[14, 64]
	Gibberellin (GA)	Phytohormone	Required for cucumber and tomato hypocotyl reunion but not inflorescence stem reunion	[19]
	ARF6 ARF8	ARF	Induced by auxin above the incision to promote pith cell division	[80]
	PIN1	Auxin transporter	Required for inflorescence stem reunion	[20]
	ANAC071	NAC	Induced by auxin and ethylene to promote cambial cell formation and tissue reconnection during inflorescence stem reunion	[20, 48]
	ANAC096	NAC	Promoting cambial cell formation and tissue reconnection during inflorescence stem reunion	[48]
	RAP2.6L	AP2/ERF	Induced by JA but inhibited by auxin and promoting cell division and tissue reunion	[64]
	XTH19, XTH20	Xyloglucan endotransglucosylase	Regulating the proliferation and elongation of pith cell to promote inflorescence stem reunion	[80]
	DOFs	DOF	Induced by auxin and cell wall damage, and required for inflorescence stem reunion	[81]

### Regulatory mechanisms of SVT regeneration after girdling

In the main, investigations of SVT regeneration after girdling are mainly carried out in woody species. Taking the advantage of high-resolution sampling strategy, genetic transformation of model tree poplar and *in vitro* SVT regeneration platform, our understanding of this regeneration process has been advanced from the histological level to cellular and molecular levels^[15,22,26,35]^.

Based on the histological observations in multiple tree species, we have learned that after the bark is removed, phloem and cambium are reconstituted from the xylem cells (Fig. 2a). To follow the transcriptomic changes during this process, cDNA microarrays were used to analyze the transcriptional profiles at different temporal stages of SVT regeneration in *Populus tomentosa*. Among the 207 differentially expressed genes (DEGs), *AUXIN/INDOLE-3-ACETIC ACID* (*Aux/IAA*) and * PIN-FORMED* (*PIN*) genes are highly expressed in the stage of cambium establishment, while the genes encoding MYB proteins and several small heat shock proteins are strongly transcribed during xylem re-differentiation^[54]^. However, such analysis used pooled regenerated tissues at each time point, no spatial changes could be obtained. To trace the tissue specific transcriptomic dynamics, a tangential cryo-section method was utilized to isolate different regenerated tissue layers at the same stage for gene profiling^[22]^. The results show that at the first stage, genes related to epigenetic regulation and cell cycle, such as DNA methyltransferases, histone acetyltransferases, chromatin remodeling-related proteins, polycomb group (PcG) proteins, cyclins and cyclin-dependent kinases (CDKs) are dominantly up-regulated, suggesting the re-entry of cell cycle of de-differentiating xylem cells, which coincides with the occurrence of cell divisions in these cells. Along regeneration, expression of xylem marker genes and xylem-specific transcription factors are significantly down-regulated, implying a loss of xylem identity. In the later steps, phloem-related and cambium-related transcription factors are up-regulated, such as phloem genes *ALTERED PHLOEM DEVELOPMENT* (*APL*) and *DNA BINDING WITH FINGER* (*DOF*) family members^[55,56]^, as well as cambial genes *AINTEGUMENTA* (*ANT*) , *KNOTTED1-LIKE HOMEOBOX GENE* (*KNOX*) and the GRAS family members *SHORT ROOT* (*SHR*) and *SCARECROW-LIKE* (*SCL*)^[57−61]^. The spatiotemporal dynamics of the expression of vascular marker genes support the anatomic characterization of new phloem SEs and cambial cells formation on the molecular level. Overall, the reported data revealed that xylem specification program is suspended while the phloem and cambium developmental programs are activated to complete the cell fates switch during SVT regeneration after girdling^[22]^. Additionally, phytohormone-related genes undergo drastic changes during all steps of phloem and cambium regeneration^[22]^.

On the protein level, using two-dimensional electrophoresis technique in combination with matrix-assisted laser desorption/ionization-time of flight mass spectrometer (MALDI-TOF MS), 244 differentially expressed proteins are identified during SVT regeneration. Proteins involved in metabolism, signaling, cytoskeleton formation and cell cycle are highly expressed in the stage of cambium re-establishment. Enzymes involved in cell wall formation are expressed in the stage of xylem re-differentiation at 18–22 DAG. For example, increased expression of INDOLE-3-ACETIC ACID INDUCIBLE 2 (IAA2) in regenerated cambium, displays that auxin might mediate cambium regeneration. Cell cycle protein PROLIFERATING CELLULAR NUCLEAR ANTIGEN (PCNA) is expressed during the whole regeneration process and this is consistent with frequent cell divisions and fate decisions during SVT regeneration. The regeneration of the xylem is also accompanied by high expression of CINNAMYL ALCOHOL DEHYDROGENASE (CAD), a key enzyme for lignin synthesis, indicating functional recovery of cambial activity to produce new xylem cells^[31]^.

In addition, miRNAs have also been reported to affect SVT regeneration partially through affecting auxin transport and signaling^[62]^. By small RNA and degradome sequencing during SVT regeneration in poplar, 21 known and 30 novel miRNA families that are dynamically expressed along SVT regeneration are identified . Among them, 15 miRNA families such as miR156, miR160, miR166, and miR171 were involved in auxin signaling, meristem initiation and organization, cell division and differentiation^[62]^. Intriguingly, several miRNAs dynamically expressed during SVT regeneration are also differentially expressed during normal SVT development^[63]^, suggesting that SVT regeneration and development share certain miRNA-mediated regulatory mechanisms.

As described above, evidences from both transcriptional and translational levels point out the potential roles of phytohormones especially auxin during vascular tissue restoration after girdling. In the last decades, researchers have explored how a variety of phytohormones regulate vascular development in plants^[21,64]^. Functional studies in trees also identify the fundamental roles of auxin, cytokinin (CK) and gibberellin (GA) for cambium development and wood formation^[65−68]^. It has been shown that during SVT regeneration in* E. ulmoides,* the content of endogenous auxin increases significantly from 2 DAG and maintain high in the whole process compared with ungirdled stem, suggesting that auxin plays an important role in the course of cell de-differentiation, trans-differentiation and re-differentiation^[69]^. The establishment of* in vitro* SVT regeneration platform makes it possible to dissect the functions of hormones without interference of endogenous source of hormones from unwounded stem parts^[26]^. Using this platform, it showed that the exogenous IAA accelerates phloem SEs trans-differentiation and cambium regeneration^[26,35]^ whereas exogenous CK alone could only promote the reconstitution of phloem SEs but not cambium^[26]^. Such influences of CK are subsequently verified in transgenic poplar lines. In the lines where CK signaling regulator *CYTOKININ INDEPENDENT-1* (*CKI1*) or biosynthesis gene *ISOPENTENYL TRANSFERASE 7* (*IPT7*) are overexpressed, phloem SEs but not cambium are formed after girdling without any hormonal treatment. On the other hand, overexpressing the CK degradation enzyme gene *CYTOKININ OXIDASE 2* (*CKX2*) exhibits fewer cambium cell divisions and less efficient phloem regeneration^[26,65]^. Intriguingly, joint auxin and CK treatment causes same consequences as CK treatment^[26]^. Tracking the changes of auxin response and distribution with DR5:GUS reveals that auxin maximum redistributes during cambium reconstruction upon auxin treatment but this does not happen on CK or auxin-CK treatments^[26]^. Transcript analysis further finds out that auxin induces the expression of *IAA3* and *ANT* leading to cambium recovery, and poplar *RESPONSE REGULATOR 7* (*RR7)* and phloem genes *APL* and *CALLOSE SYNTHASE 7* (*CALS7)* leading to phloem formation*.* Differently, CK promotes the expression of *RR7* and phloem genes but inhibits* ANT* thus the cambium formation^[26]^ (Fig. 2b). All the data unveil the differential roles of auxin and CK during SVT regeneration.

### Regulatory mechanisms of root tip regeneration after excision

The regulatory network of root tip regeneration is the most comprehensive among the plant *in situ* regeneration systems. A number of molecular regulators including different phytohormones and transcription factors and the hierarchy relations among them have been identified in root tip regeneration (Fig. 2d). Root tip excision leads to the redistribution of auxin and CK locally. In the uncut root tip, auxin is mainly localized in columella, QC and stele, while CK is mainly localized in LRC and columella^[70]^. When the root tip is removed, auxin and CK overlap at the wound transiently and then separate, resulting in a proximal CK and distal auxin distribution. External application of auxin or CK changes their domains and thus the position of stem cell niche^[17]^. The accumulation of auxin at the root tip activates the key transcription factor *AUXIN RESPONSE FACTOR* 5/*MONOPTEROS* (*ARF5/MP*), thereby promoting the reconstruction of root meristems^[9,17,42]^. It has been noticed in different plants that the root tip cannot regenerate when the cut is higher than a certain position, instead, new lateral roots will form in this situation^[40,42]^. Later on, it is found that the regenerative ability of the root tip depends on the expression patterns of transcription factor *PLETHORA 2* (*PLT2*). The competence zone in RAM with high *PLT2* expression has a strong regenerative ability and the beyond-competence zone with low *PLT2* expression has a weak regenerative ability. Transient overexpression of *PLT2* confers the beyond-competence zone regeneration ability whereas sustained overexpression of *PLT2* beyond a threshold or downregulation of *PLT2* in competence zone would inhibit its ability to regenerate^[71]^. The fact that auxin could restore the ability of regeneration in high-cut roots indicates that the loss of regenerative ability in the beyond-competence zone could be due to the destruction of the auxin sources. This hypothesis is further supported by the study in which, root could not regenerate in the auxin synthetase YUCCA-deficient quintuple mutant *yuc3 yuc5 yuc7 yuc8 yuc9* (*yucQ*) and auxin synthesis inhibitor l-kynurenine treatment^[72]^. Recent investigations disclosed the roles of jasmonic acid (JA) and the downstream transcription factors in root tip regeneration^[16,70,73]^. Both root tip excision and QC ablation induce rapid elevation of JA and auxin in the wound^[16]^. Increased JA promotes bHLH family transcription factor MYC2 directly binds and regulates transcription factors *ETHYLENE RESPONSE FACTOR 109* (*ERF109*) and *ERF115.* ERF109 activates *CYCLIN D6;1* (*CYCD6;1*) and together they act upstream of *ERF115*, which is required for root tip regeneration^[16]^. As a core regulator of root tissue regeneration, ERF115 can interact with stem cell regulating module SHORTROOT-SCARECROW-RETINOBLASTOMA-RELATED (SHR-SCR-RBR)^[16]^ and with PHYTOCHROME A SIGNAL TRANSDUCTION1 (PAT1) to induce the expression of *WOUND INDUCED DEDIFFERENTIATION1* (*WIND1*) and the peptide* PHYTOSULFOKINE 5* (*PSK5*), thereby to regulate the reconstruction of stem cell niche and the root tip^[73,74]^. Interestingly, the local auxin synthesis in root is inhibited in *erf115* mutant, indicating the interaction between JA and auxin pathways during root tip regeneration^[72]^. A recently published study found that *ERF114*, the homolog of *ERF115*, is strongly expressed at 5 h after root tip excision. And overexpression of *ERF114* could enhance the root sensibility to auxin and positively regulate regeneration. Besides, the expression of *ERF114* and *ERF115* is activated directly by a central component of the brassinosteroid (BR) signaling pathway but inhibited by BZR1 and FERONIA (FER)^[75]^.

As described in the above session, using laser ablation, cells with a specific cell identity such as QC and stem cells can be eliminated in root tip. With a set of cell identity markers and hormonal markers in addition to relevant mutants, the molecular mechanisms have been unraveled. It is found that auxin accumulates at the wound after QC cells are ablated, leading to the up-regulation of *PLT* expression as well as the downregulation of *PIN* expression. PLT promotes nuclear localization of SHR, which in turn induces *SCR* expression. Together, SCR and PLT regulate correctly polarized localization of PIN and reconstruction of QC cells^[76]^. Generally, when one type of cells is abolished, the cells on the inside of the ablated cells can replace them. However, ablations of different cells may reactivate specific regulation modules involved in the reconstruction process. For example, when LRC cells are ablated, FEZ/SOMBRERO (FEZ/SMB) module is induced in the adjacent epidermis cells to reconstruct new LRC; when cortex cells are ablated, SHR/SCR-CYCD6;1 module is activated in the near endodermal cells to reform new cortex layer^[47]^. What's more, auxin receptor TRANSPORT INHIBITOR RESPONSE 1/AUXIN-SIGNALING F-BOX proteins (TIR1/AFBs)-mediated auxin signaling activates the expression of *ERF115* and promotes cell proliferation and expansion after cell ablation^[46]^, and ERF115, in turn, activates *ARF5* to drive root stem cell regeneration. These results indicate that the root tip regeneration and root specific cell regeneration share some regulatory modules.

### Regulatory mechanisms of tissue reunion after incision

Although auxin is involved in nearly all tissue regeneration systems, it might be less important for tissue reunion after partial incision of hypocotyl in cucumber and tomato seedlings^[14]^. On the other hand, it has been noticed that cotyledon and root play important roles during the wound healing in cucumber and tomato hypocotyl after partial incision. Later it has been demonstrated that it is the GA from cotyledon and microelements such as boron, manganese and zinc ions from roots that are required for this tissue reunion process^[77]^. After incision of cucumber or tomato hypocotyls, GA is required for cortical cell division, as cortex cell division and successive hypocotyl reunion are not observed in tomato GA-deficient mutant *gib-1*. In addition, removing cotyledons of the seedling leads to the failure of hypocotyl restoration and applying GA3 to shoot apex could reverse the inhibitory effects. However, this effect could not be replaced by IAA, and hypocotyl tissue reunion could occur normally even if auxin polar transport is inhibited, suggesting that GA rather than auxin is required for hypocotyl reunion^[19]^. What’s more, root derived microelements are found to be essential for intrusive cell elongation during tissue reunion in the cortex of cucumber hypocotyls^[78]^. If the root is removed, cortex cells could divide normally but could not elongate to form tight connection after hypocotyl incision^[78]^. Intriguingly, different from the above systems, auxin and especially perception of auxin seems to play a pivotal role during hypocotyl graft reunion in Arabidopsis^[3]^. So it would be interesting to further explore on the mechanistic differences between the partial incision and grafting of hypocotyl. The regulatory mechanism underlying stem reunion might be also different from that of cucumber and tomato hypocotyl reunion. The reunion of incised inflorescence stem is not affected in the GA-deficient Arabidopsis mutant *gibberellin 3-oxidase 1/gibberellin 3-oxidase 2* (*ga3ox1/ga3ox2*) but dramatically inhibited in *pin1* mutant. These results indicate that auxin rather GA participates in the reconnection of the inflorescence stem in Arabidopsis^[20]^. Similarly, auxin around the incision promotes the formation of cambium and induces cambium cells differentiation into xylem and phloem in tobacco stem^[79]^. In fact, the vascular tissue regeneration after incision is proven to be guided by the auxin 'canalization'^[49]^. Temporal and spatial analyses revealed that in incised Arabidopsis stems, induction of cambium-like cells and emergence of new vasculature is correlated with reorganized auxin response and auxin polarity. Characterization on the molecular markers of cambium and xylem, including TDIF RECEPTOR/PHLOEM INTERCALATED WITH XYLEM (TDR/PXY) and ARABIDOPSIS THALIANA HOMEOBOX8 (ATHB8) during stem reunion further supports the functions of auxin in vascular reconnection^[48,49,52]^.

Another noteworthy mechanism that operates the tissue reunion after incision in stem is related to the asymmetric response in the upper and lower regions of the incision site (Fig. 2e & f). For instance, auxin accumulation is higher above the wound and lower below the wound due to the block of auxin polar transport. In the upper region of incision, the auxin promotes the expression of *NAC DOMAIN CONTAINING PROTEIN71* (*ANAC071*) via ARF6 and ARF8, and *ANAC071* can also be activated by ethylene^[20]^. Whereas in the lower region of incision, the lower auxin level together with JA promotes the expression of *RELATED TO AP2.6L* (*RAP2.6L*) via the induction of DEFECTIVE IN ANTHER DEHISCENCE 1 (DAD1)^[14,77]^. In addition, ANAC071 induces the expression of *XYLOGLUCAN ENDOTRANSGLUCOSYLASE*/*HYDROLASES 19* (*XTH19*) and *XTH20* to regulate the proliferation and elongation of pith cell^[64,80]^. Recent report also identified that ANAC071 and its homolog ANAC096 redundantly regulate vascular tissue reunion, and they are necessary for the formation of cambium-like cells^[48]^. What’s more, the DOF family transcription factors are activated by auxin accumulation soon after stem incision. And quadruple *hca2 tmo6 dof2.1 dof6* (*dofQ*) mutant shows impaired wound healing after inflorescence stem incision^[81]^. It is worth mentioning that the incision also induces the expression of phloem-related genes *SIEVE ELEMENT OCCLUSION-RELATED 1* (*SEOR1*), xylem-related genes *VASCULAR RELATED NAC-DOMAIN PROTEIN 7* (*VND7*) and* XYLEM CYSTEINE PEPTIDASE 1* (*XCP1*) as well as the cambium-related genes *WUSCHEL RELATED HOMEOBOX 4* (*WOX4*). These data suggest that the activation of vascular tissue development programs is important for inflorescence reunion after incision^[48]^.

## Comparisons of plant *in situ* tissue regeneration systems

Plants have extraordinary capacity to regenerate, ranging from wound repair of specific organs, tissues or even cells to an absolute new organism. Plant *in situ* regeneration refers to the former scenario. Herein before, we described the regenerative dynamics and the regulatory mechanisms underneath different *in situ* tissue regeneration processes during both primary growth and secondary growth of plants. A couple of common features are recognized by comparing SVT regeneration after bark girdling, apex regeneration after excision and tissue reunion after incision (Fig. 2).

First, the regenerative responses are similar in the three regeneration systems. All plant *in situ* tissue regeneration begin with the perception of stimulation from wound or damage, which triggers cell cycle re-entry of remaining or surrounding cells. The regeneration processes involve loss of previous cell identities and gain of new cell identities *via* gene expression reprogramming to complete the cell fate transformation. The final tissue restoration also involves cell re-differentiation to the cell types that are damaged or lost before.

Second, meristem or meristem-like tissue reconstitution and vascular repatterning occur in all three systems. However, a functional stem cell niche might not be required for tissue regeneration. After cutting of Arabidopsis root tip, other cell types could recover earlier than QC cells and they can re-differentiate directly from neighboring cells without restoring to the stem cell state^[17,42]^. Similarly, in SVT regeneration, immature secondary xylem cells are eligible to trans-differentiate into phloem SEs preceding the resumption of a functional vascular cambium^[26,35]^. Vascular tissue re-establishment is part of tissue regeneration in all three systems although in root tip, only primary vascular tissues are restored whereas in the other two systems, SVT reconstruction could be included and therefore, the regeneration of cambial or cambium-like activity is essential.

Third, in *in situ* regeneration, the regenerative capacities of related cells are limited, which is closely related to their developmental status^[82]^. For instance, trees with more vigorous secondary xylem have a stronger ability to regenerate SVT after girdling. Likewise, the root tip has the regenerative ability only when excision is performed within the region expressing *PLT* in the root, that is the competence zone^[71]^. This phenomenon is also common in other regeneration processes, for example, young leaves have a higher regeneration efficiency than older leaves^[42]^.

Furthermore, phytohormones are important regulators of plant *in situ* tissue regeneration and auxin plays a central regulatory role in all three tissue regeneration systems. The application of auxin can promote both root tip regeneration and SVT regeneration after girdling, while inhibition of auxin polar transport by 1-N-naphthylphthalamic acid (NPA) suppresses the reconstruction of a new root tip as well as a new bark comprising SVTs^[26,42]^. Although auxin is not essential in hypocotyl reunion in cucumber and tomato^[14,19]^, auxin polar transport is required for Arabidopsis inflorescence stem reunion^[20,52]^. Since only limited varieties of phytohormones are inspected in each *in situ* regeneration system, it would be very interesting and important to test certain hormone and the downstream transcription factors that are identified in other systems but have not yet been examined.

## Conclusions and future perspective

### Unsolved questions in the study of plant *in situ* regeneration

As described earlier in this review, we now have an advanced understanding of plant *in situ* regeneration on multiple levels. However, there are still many questions that deserve further discussion. First, what are the key signals that initiate plant *in situ* regeneration? It is proposed that reprogramming of cells in the injured region is ectopically activated by the integration of intrinsic and extrinsic signals^[46]^. Previous research has focused on phytohormone-centered signals in response to injury, but how other signals including, chemical signals like reactive oxygen species (ROS), electrical signals as calcium spikes as well as mechanical forces, regulate plant *in situ* tissue regeneration remain unclear^[9]^. Second, how do cells around the wound recognize the signals and fulfill the identity transition? Most studies on *in situ* regeneration tend to consider the related tissue or organ as a whole, but recent work demonstrated that regenerated tissues usually originate from relatively small cell populations in the damaged area^[23]^. Therefore, it is of value to identify these cell populations through high-resolution imaging or single-cell techniques and study how external signals change the cell fate of these cells. Third, what are the similarities and differences in the regulatory mechanisms between plant regeneration after injury and normal development? Some studies have identified many regulators that are only induced in injury responses, whereas some found a number of regulators and signaling pathways that are shared in tissue regeneration and normal developmental programs^[8,83]^. Take SVT regeneration as an example, multiple factors regulating SVT development also play roles during SVT regeneration. However, during SVT regeneration, the normal developmental programs are changed dramatically for vascular tissues through de-differentiation and re-differentiation. For example, phloem appears before functional cambium formation during SVT regeneration, which doesn't occur during natural SVT development^[22,26,35]^. What’s more, while CK could promote phloem development and regeneration, it plays a different role in the development and regeneration of cambium. It has been demonstrated that CK stimulates the meristematic activity of the established cambium and enhances auxin concentration and response in cambium during the normal SVT development, however, it appears inhibiting the recovery of cambium and suppressing auxin maximum reformation during SVT regeneration^[26]^. Therefore, efforts to distinguish and characterize these aspects will benefit us in many ways, for instance, designing new crops or trees with high tissue or organ regenerative capacity without interfering the normal developmental trends.

### From Arabidopsis to forestry trees

To date, our understanding of plant *in situ* tissue regeneration mainly comes from the studies of Arabidopsis, but there might be different mechanisms in other species. Meanwhile, improving woody plant regenerative ability has always been an important topic in forestry research as for tissue culture, genetic transformation and the vegetative propagation of trees. Since various regeneration processes between Arabidopsis and trees are similar, for example, tissue reconnection occurs in both Arabidopsis stem repair and forest grafting, it is natural to wonder if they share regulatory mechanisms. In forestry and horticulture production, tissue damage occurs frequently, such as root breaking during transplanting^[84]^, mechanical damage to stems that induces changes in wood structure and quality^[85,86]^. Like Arabidopsis, woody plants respond to wounds and initiate *in situ* regeneration procedures. Little is known about tissue repair in response to wounding in woody species. Moreover, how do the key factors, functioning in Arabidopsis, affect tree regeneration and development remain unexplored. Therefore, the identification of key regulators in Arabidopsis such as PLTs^[76]^, WIND1^[87,88]^, ERF115^[73,74,89]^, ANAC071 and RAP2.6L^[20,90,91]^ gives us the best opportunities to explore the functions of these regulators in different regeneration processes in forestry trees (Fig. 2). Approaches commonly used in Arabidopsis, for instance, the tissue specific ablation method, linage-tracing analysis and advanced imaging protocols can be translated to trees to obtain a more comprehensive regulatory framework for SVT regeneration and other forestry related regeneration such as stem cutting and grafting.

### Future perspective for forestry research

Plant *in situ* regeneration is not only an important way for plants to survive after wounding, it is also a promising tool for horticulture and the forestry industry. Therefore, unraveling the mechanisms of *in situ* regeneration is of great significance to forestry research through generating new tree genotypes with stronger regenerative capacity and better traits^[12,92]^. Together with the utilization of advanced techniques on gene editing, genetic transformation, pan-genome analyses, scRNA-seq and our increasing knowledge on wood formation in trees, the SVT regeneration system will become a powerful tool in forestry research. Further verification of known regulators uncovered in other *in situ* regeneration systems and in other plants as well as identification of novel factors in forestry trees will enable us to exploit new targets for scientific research on vascular development and for molecular breeding to improve wood yield and quality.
